# A Topological Cluster of Differentially Regulated Genes in Mice Lacking PER3

**DOI:** 10.3389/fnmol.2020.00015

**Published:** 2020-02-13

**Authors:** Daan R. Van der Veen, Emma E. Laing, Sung-Eun Bae, Jonathan D. Johnston, Derk-Jan Dijk, Simon N. Archer

**Affiliations:** ^1^School of Biosciences and Medicine, Faculty of Health and Medical Sciences, University of Surrey, Guildford, United Kingdom; ^2^UK Dementia Research Institute, London, United Kingdom

**Keywords:** mouse, circadian, topological associating domain, metabolism, transcriptome

## Abstract

Polymorphisms in the human circadian clock gene *PERIOD3* (*PER3*) are associated with a wide variety of phenotypes such as diurnal preference, delayed sleep phase disorder, sleep homeostasis, cognitive performance, bipolar disorder, type 2 diabetes, cardiac regulation, cancer, light sensitivity, hormone and cytokine secretion, and addiction. However, the molecular mechanisms underlying these phenotypic associations remain unknown. *Per3* knockout mice (*Per3^–/–^*) have phenotypes related to activity, sleep homeostasis, anhedonia, metabolism, and behavioral responses to light. Using a protocol that induces behavioral differences in response to light in wild type and *Per3^–/–^* mice, we compared genome-wide expression in the eye and hypothalamus in the two genotypes. Differentially expressed transcripts were related to inflammation, taste, olfactory and melatonin receptors, lipid metabolism, cell cycle, ubiquitination, and hormones, as well as receptors and channels related to sleep regulation. Differentially expressed transcripts in both tissues co-localized with *Per3* on an ∼8Mbp region of distal chromosome 4. The most down-regulated transcript is *Prdm16*, which is involved in adipocyte differentiation and may mediate altered body mass accumulation in *Per3^–/–^* mice. eQTL analysis with BXD mouse strains showed that the expression of some of these transcripts and also others co-localized at distal chromosome 4, is correlated with brain tissue expression levels of *Per3* with a highly significant linkage to genetic variation in that region. These data identify a cluster of transcripts on mouse distal chromosome 4 that are co-regulated with *Per3* and whose expression levels correlate with those of *Per3*. This locus lies within a topologically associating domain island that contains many genes with functional links to several of the diverse non-circadian phenotypes associated with polymorphisms in human PER3.

## Introduction

The circadian timing system defines biological timing and its synchronization to the environmental 24-h light/dark cycle. The central pacemaker in the suprachiasmatic nuclei (SCN) of the hypothalamus entrains to environmental cues and synchronizes peripheral clocks in tissues and organs to regulate 24-h rhythms in physiology and behavior. A set of highly conserved circadian clock genes and their products interact in the molecular feedback loops that determine circadian clock function (Archer and Oster, 2015).

The *Period3* (*Per3*) gene is a component of the circadian clock. Its presence is not required for central circadian clock function, although absence of PER3 in mice has subtle effects on the circadian period of locomotor activity, and larger effects on the period of circadian gene expression in *ex vivo* dissociated SCN cells and peripheral organ cell cultures (Shearman et al., 2000; Bae et al., 2001; Pendergast et al., 2012; Ramanathan et al., 2014). Absence of functional PER3 protein in *Per3* knock out mice (*Per3^–/–^*) has also been associated with reduced inhibition by light of locomotor activity (negative masking), and changes in the overall amount of locomotor activity and sleep homeostasis (van der Veen and Archer, 2010; Hasan et al., 2011).

We have shown that a primate-specific, variable number, tandem repeat (VNTR), coding region polymorphism in human *PER3* is associated with diurnal preference, delayed sleep phase disorder, sleep homeostasis, increased body mass index (BMI) in people who sleep late during work days, cognitive performance, fMRI-assessed brain activity, lower intelligence in people with long sleep duration during work days, and cardiac regulation (Archer et al., 2003; Viola et al., 2007, 2012; Groeger et al., 2008; Vandewalle et al., 2009, 2011; Lazar et al., 2012; Lo et al., 2012). Others have reported associations with bipolar disorder (Benedetti et al., 2008), schizophrenia (Karthikeyan et al., 2014a), white matter integrity (Bollettini et al., 2017), type 2 diabetes (Karthikeyan et al., 2014b), cancer (Alexander et al., 2015), light sensitivity (Chellappa et al., 2012), hormone secretion (Wirth et al., 2013), cytokine secretion (Guess et al., 2009), and addiction (Zou et al., 2008). In addition, we have also shown that transgenically humanizing mouse *Per3* with the VNTR polymorphism phenocopies some aspects of human sleep homeostasis such as increased theta power during wake and delta power during sleep (Hasan et al., 2014). Thus, PER3 is associated with a wide range of phenotypes, but the molecular mechanisms underlying these pleiotropic effects remain unknown.

Because *Per3* is a circadian clock gene that has been associated with light-dependent phenotypes, here we characterized differential gene expression between wild type and *Per3^–/–^* mice in hypothalamic and whole eye tissue during an ultradian light/dark protocol that produced different behavioral responses in the genotypes. Many transcripts that were differentially expressed in *Per3^–/–^* mice localized to a cluster of genes around *Per3* on distal chromosome 4. Expression quantitative trait loci (eQTL) analyses identified strong linkage between *Per3* expression and genetic variation within the same distal chromosome 4 cluster and high correlation between *Per3* expression and the expression of genes within the cluster. Differentially expressed transcripts were related to non-circadian functions including sleep homeostasis and identify genetic targets that might underlie some of the diverse phenotypes associated with *PER3*.

## Materials and Methods

### Animals

Male *Period3* knockout mice (*Per3^–/–^*) (Shearman et al., 2000) and their wild-type (WT) litter mates on a C57Bl/6J background were bred at the University of Surrey, as described previously (van der Veen and Archer, 2010). Genotyping was performed as previously described (Shearman et al., 2000). Throughout all experiments, mice were kept under controlled environmental conditions [20–22°C ambient temperature, 55 ± 10% relative humidity, light intensity at cage bottom was ∼170 mW/m^2^ (∼50 lux)]. All experimental procedures were approved by the University of Surrey Animal Ethics Committee and carried out under a United Kingdom Home Office License.

### Animal Experiments

To investigate differential gene expression between WT and *Per3^–/–^* mice, animals were subjected to an ultradian light/dark paradigm that we have previously published (van der Veen and Archer, 2010). Male, adult WT and *Per3^–/–^* mice [*N* = 8 per genotype, age = 66.0 ± 3.4 days (mean ± SEM)] were individually housed in running wheel cages in a 12 h light/12 h dark cycle (LD 12:12) for 10 days. Mice were subsequently exposed to 17 cycles of an LD 3.5:3.5 starting at lights-on on day 11. In the second hour of the last ultradian light cycle, mice were killed by cervical dislocation and tissue (brain and whole eye) was collected in RNAlater stabilization reagent (Qiagen, United Kingdom) and frozen at −80°C until further analysis. Running wheel data were collected throughout the experiment (ClockLab, Actimetrics, Wilmette, IL, United States) and used to confirm previously observed significant attenuation of behavioral suppression by light in *Per3^–/–^* mice (van der Veen and Archer, 2010).

To investigate genotype differences in body mass accumulation, normal diet-fed male WT and *Per3^–/–^* mice were individually housed under LD 12:12 at 4 weeks of age [28.0 ± 0.9 days (mean ± SEM)] and switched to either a 10 kcal% (control) or 45 kcal% (high) fat diet (D12450B and D12451, respectively, Research Diets, Inc., NJ, United States). The LD 12:12 cycle was advanced by 4 h every 7 days (Davidson et al., 2006), and this protocol was continued for 14 weeks. Body mass and food consumption was established twice per week (*N* = 8 per genotype, per diet). Body mass data for two WT mice on high fat diet was removed from the analysis due to instability of body mass during the protocol. Data was statistically tested using Proc MIXED in SAS (Cary, NC, United States).

### Tissue Specific Differential Gene Expression

The whole-genome transcriptome was quantified by microarrays as previously described (Hasan et al., 2014). In short, for both *Per3^–/–^* and WT mice (*N* = 8 per genotype) mRNA was purified from a frozen block of hypothalamic tissue and whole eye using the RNeasy Mini Kit (Qiagen, United Kingdom). Hypothalamic blocks were prepared by placing the frozen brain on ice, and then ventral to dorsal cutting a square (∼2 × 2 mm) boxed around the optic chiasm in the horizontal plane [starting at 0.02 mm caudal to Bregma in Paxinos and Franklin (2001)], trimmed to a depth of ∼2 mm. This block included the SCN of the hypothalamus. RNA quantity was measured spectrophotometrically (Nanodrop, Thermo Fisher Scientific, Waltham, MA, United States) and integrity was quality controlled using an Agilent Bioanalyzer (Agilent Technologies United Kingdom Limited, United Kingdom). RNA was used to prepare labeled cRNA, which was hybridized to Agilent Whole Mouse Genome slides (60 mer, 4 × 44K features), and array features were extracted using Agilent Feature Extraction Software (Agilent Technologies). The results of these array data have been made publically available at the NCBI Gene Expression Omnibus database. Expression levels were analyzed using GeneSpring version 12 (Agilent Technologies). Differential expression of probes and genes was quantified both as the fold change expression between genotypes, and significantly differential expression between genotypes were identified using unpaired *T*-Tests with an asymptotic *P*-value computation with Benjamini–Hochberg correction for multiple testing. As a second approach to identify differential gene expression between WT and *Per3^–/–^* mice, we applied the RankProdIt analysis, as described previously (Laing and Smith, 2010). One sample for *Per3^–/–^* whole eye was removed due to hybridization errors.

### Quantitative PCR

Male, adult WT and *Per3^–/–^* mice [*N* = 8 per genotype, age = 65.0 ± 1.6 days (mean ± SEM)] were individually housed in running wheel cages in an LD 12:12 for 19 days, after which they were exposed to constant darkness. On the second day of constant darkness (DD), three mice per genotype were culled every 4 h at circadian time (CT) 0, 4, 8, 12, 16, and 20 (CT0 = time of previous lights on). Mice were killed by cervical dislocation and tissues were harvested in RNAlater stabilization reagent (Qiagen, United Kingdom) and frozen at −80°C until further analysis. RNA was extracted using the RNeasy Mini Kit (Qiagen, Hilden, Germany) according to the manufacturer’s instructions. For each sample, the relative levels of expression of *Prdm16* and housekeeping gene large ribosomal protein subunit P0 (*Rplp0*) were measured using Taqman qPCR and quantified using the delta delta CT method. The qPCR primers and probe for each gene are shown in Supplementary Table S1.

### Expression Quantitative Trait Loci Analyses

eQTL were computed with the use of GeneNetwork^1^ (Mulligan et al., 2017). In GeneNetwork there are six record ID options to select that target different regions of *Per3* mRNA. Gene Network recommends selecting a record ID that targets an exonic region so for the analyses presented here, we selected record 1421086_at, which targets the last three exons and proximal 3′ UTR of *Per3*. eQTLs for *Per3* in the mouse BXD RI family were mapped for Brain mRNA [UCHSC BXD Whole Brain M430 2.0 (Nov06) RMA (Saba et al., 2006)], Eye mRNA [Eye M430v2 (Sep08) RMA], and Liver mRNA [GSE16780 UCLA Hybrid MDP Liver Affy HT M430A (Sep11) RMA (Bennett et al., 2010)]. Using the GeneNetwork mapping tool, we computed the likelihood ratio statistics (LRS score) for 1421086_at using interval mapping (1000 permutations). We next computed the sample correlations to establish the top 500 correlated (Pearson’s *R*) expression regions of 1421086_at for the eye mRNA dataset, and the chromosomal locations of the their maximal LRS locations. We established the gene location of the specific transcripts that have a maximal LRS in one of five chromosomal regions with maximal LRS counts > 50, and for each of the five groups calculated whether the number of linked genes per chromosome were more, or less than expected: Expected per chromosome = (# genes chromosome/# genes genome) × # genes in cluster; Fraction of expected = (observed per chromosome/expected per chromosome) − 1.

### Bioinformatics

Gene ontology (GO) enrichment for biological processes and molecular functions associated with gene expression data was performed using Webgestalt 2019 (Zhang et al., 2005; Liao et al., 2019). We performed over representation analysis with the geneontology database using mouse gene symbol annotation and the mouse genome as a reference. *P* values were Benjamini–Hochberg corrected and a false discovery rate (FDR) of <0.05 was used to select significant terms. Gene/protein interaction networks for differentially expressed genes were generated using STRING v11 with the confidence of interaction set to “high.” Unconnected nodes in the generated networks were not displayed. For the topologically associating domain (TAD) analysis, we used the Hi-C browser at Penn State University^2^. Mouse assembly mm9 was used together with mouse cortex Hi-C data (Dixon et al., 2012) at a resolution of 40 kb.

## Results

### Differential Gene Expression in the Eye and Hypothalamus of *Per3^–/–^* Mice

**FIGURE 1:**
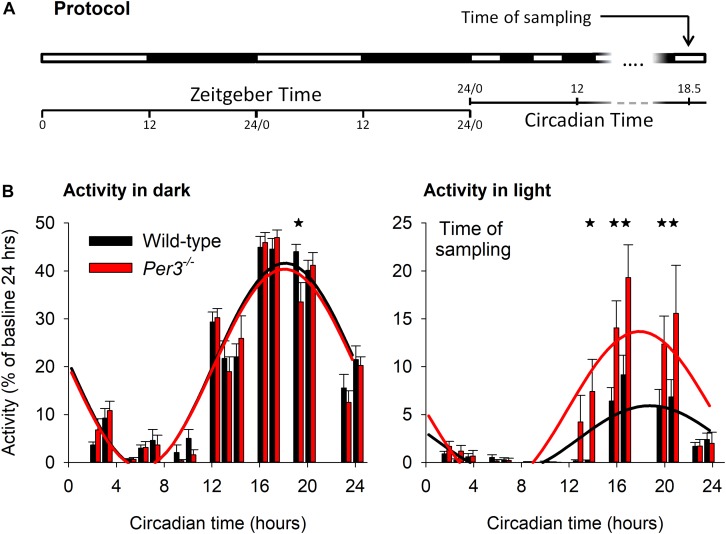
Ultradian light/dark protocol and activity behavior. Light-dark protocol of *Per3^–/–^* and WT mice **(A)**, indicating that mice were killed in the 17th ultradian light-dark cycle, corresponding to circadian time (CT) 18.5. **(B)** Group mean total behavioral activity in the dark (left) and light (right) as percentage of total activity in baseline during ultradian light-dark cycles, as a function of CT. Cosinor fits to activity (WT = black, *Per3^–/–^* = red) indicated significantly higher levels of activity during the light phase for *Per3^–/–^* mice, with maximal predicted differences in negative behavioral masking at the time of sampling. Please note that all asterisks are single but several adjacent time points are significant at the *P* < 0.05 level.

To investigate the contribution of *Per3* to molecular pathways that could underlie some of its diverse associated phenotypes, we performed transcriptomic analysis of hypothalamic and whole eye tissue from WT and *Per3^–/–^* mice. When mice are exposed to light during their active dark period, they reduce locomotor activity (negative masking) and we have previously shown reduced negative masking in *Per3^–/–^* mice compared to WT when exposed to an ultradian light/dark protocol (3.5 h light/3.5 h dark) (van der Veen and Archer, 2010). We therefore collected tissue samples from mice on the same ultradian protocol. Mice were kept on an LD 12:12 cycle for 10 days and then exposed to 17 cycles of LD 3.5:3.5. Tissues for transcriptomics were collected during the 17th ultradian light cycle, which corresponded to the prior circadian mid-active phase, a time when we previously saw the greatest behavioral difference between genotypes (Figure 1A). Analysis of wheel-running activity during the ultradian protocol (Figure 1B) confirmed our previous observation of reduced suppression of activity in *Per3^–/–^* mice during the ultradian light periods compared with WT (*P* < 0.001 for genotype^∗^time, *post hoc P* < 0.05).

Transcriptome analyses identified 74 probes in the hypothalamus (mapping to 67 annotated transcripts) that were differentially expressed > twofold between the genotypes, of which 35 were upregulated and 39 were downregulated (Supplementary Data File S1). In the eye, there were 75 probes (mapping to 71 annotated transcripts) that were differentially expressed > twofold between the genotypes (28 upregulated, 47 downregulated) (Supplementary Data File S1). By far the most down-regulated (>sevenfold) transcript in both hypothalamus and eye in *Per3^–/–^* was *Prdm16* (PR/SET domain 16), a transcriptional regulator that functions in adipose cell differentiation (Seale et al., 2007), neurogenesis (Baizabal et al., 2018), and transforming growth factor beta (TGFβ) signaling (Zeng et al., 2017).

Large average fold changes in expression may also have large variance in expression values and therefore insignificant *p* values and ranking probes according to corrected *p* values identified fewer differentially expressed transcripts. Conversely, small variance in expression values can give rise to significant *p* values but small associated fold changes. Thus, either approach applied on its own can fail to detect biologically relevant differential gene expression. We therefore also applied an approach that combines fold change and significance levels to determine differentially expressed gene ranking (Xiao et al., 2014).

**FIGURE 2:**
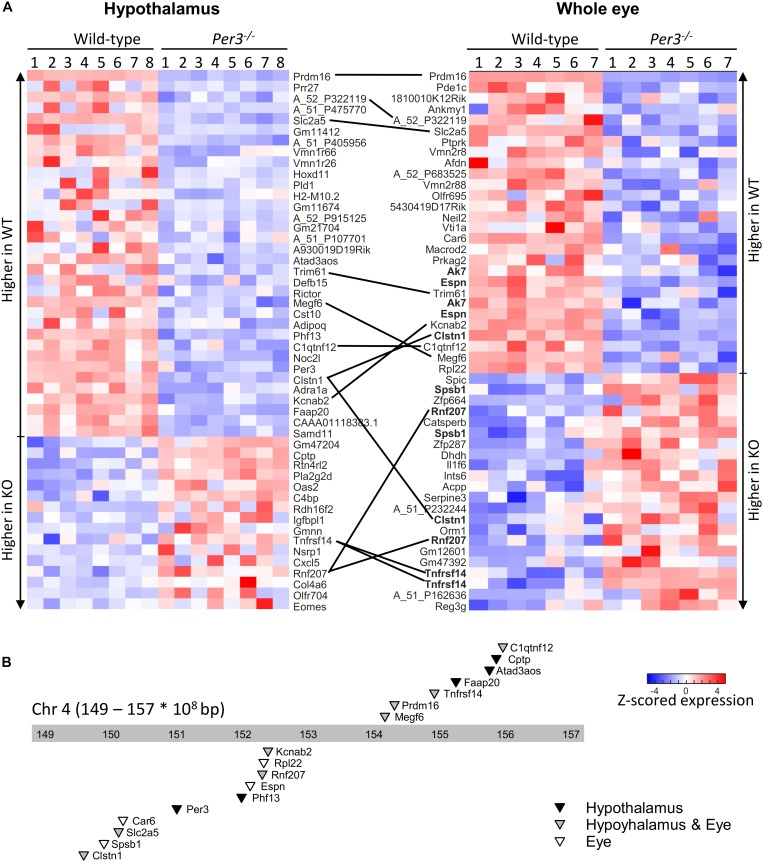
Differential gene expression between WT and *Per3^–/–^* mice. **(A)** Top-50 differentially regulated microarray probes in the hypothalamus and whole eye of WT, and *Per3^–/–^* mice. Each row depicts normalized transcript expression in biological replicates (*N* = 8 per genotype for hypothalamus, and *N* = 7 per genotype for whole eye), and are top-to-bottom ordered from largest to smallest fold-change between WT and *Per3^–/–^* mice, in groups of up- and down-regulated probes. Probes – or where possible associated gene names of probes – are indicated, and black lines connect the same probes/genes between tissues. Multiple probes targeting the same gene in the eye are in bold. **(B)** Chromosome location of 17 genes represented in the probes from **(A)**, which are all localized around *Per3*, on the distal end (149–157 × 10^8^bp) of mouse chromosome 4. Triangles point to loci of the probes, and shading indicates whether genes were differentially regulated in the hypothalamus (black), whole eye (white), or both (gray).

The top 50 differentially expressed transcripts identified from this combined analysis for each tissue are shown in the heatmaps of Figure 2A (also Supplementary Data File S2) where expression values are shown for all individual mice. This analysis identified a larger overlap of differentially expressed transcripts between the tissues (13 transcripts in common) and also highlighted multiple transcripts for the same gene being differentially expressed in the eye. Twenty-three of the transcripts that code for 17 known genes are co-located in an ∼8 Mb region (149.000–157.000 Mbp) surrounding *Per3* on mouse distal chromosome 4 (Figure 2B) and the majority of these are downregulated in *Per3^–/–^* mice, including *Per3* itself. These data suggest that the expression levels of genes within this co-located cluster are co-regulated with *Per3* and predominantly in a positive way.

Differentially expressed transcripts co-localized with *Per3* were associated with glucose/fructose regulation (*Slc2a5, C1qtnf12*), phospholipase activity (*Cptp*), RNA binding and translation (*Rpl22*), DNA repair (*Faap20*), potassium channel and cardiovascular function (*Kcnab2*, *Rnf207*), chromatin modification (*Prdm16*, *Phf13*), and immune response (*Spsb1*, *Tnfrsf14*). Some are also involved in diseases such as Alzheimer’s (*Clstn1*) and deafness (*Espn*). If probes are simply ranked according to uncorrected *p* value, this co-localization enrichment increases to include 17 and 14 of the top-20 probes for the hypothalamus and eye, respectively, and includes additional transcripts of interest such as *Dvl1* (Wnt signaling) and *Park7* (Parkinson’s disease).

**FIGURE 3:**
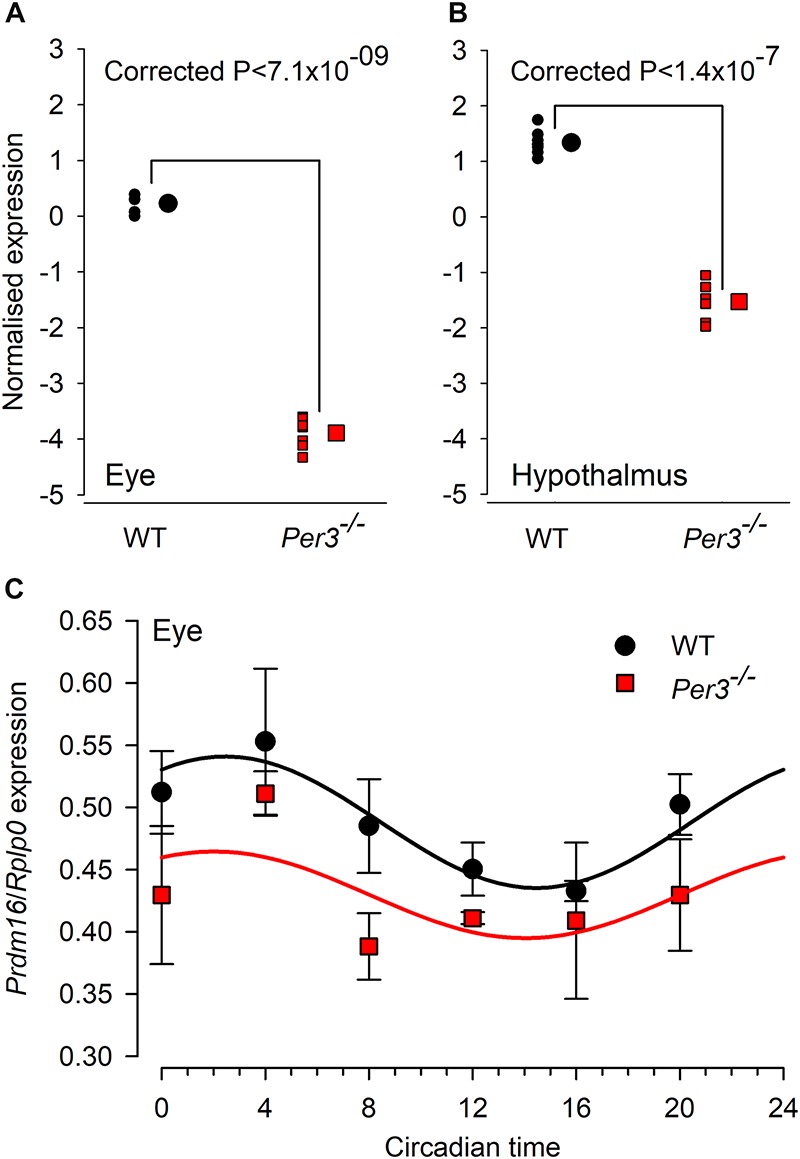
Individual microarrays and qPCR confirmation for differential *Prdm16* expression. Normalized microarray expression levels of *Prdm16* mRNA in whole eye **(A)** and hypothalamus **(B)** of individual wild-type (black circles) and *Per3^–/–^* (red squares; *N* = 7 per genotype). Large black circles and red squares are the mean (±SEM, not visible) normalized expression levels for WT and *Per3^–/–^*, respectively. **(C)** *In vivo* circadian expression patterns of *Prdm16* mRNA (mean ± SEM) was confirmed through qPCR analysis in mouse whole eye, sampled across the circadian day (*N* = 8 per genotype). *Prdm16* mRNA exhibited circadian expression patterns in both WT and *Per3^–/–^* mice, but overall levels in *Per3^–/–^* mice were significantly lower (*T*-test, *P* < 0.05).

The combined fold change/*p* value analysis confirmed *Prdm16* to be the most down-regulated transcript in both tissues. When the expression levels of *Prdm16* in both eye and hypothalamus are compared for individual WT and *Per3^–/–^* mice, the very tight clustering of expression values demonstrates the consistency of this effect (Figures 3A,B). We also observed the down regulation of *Prdm16* by qPCR amplification in samples of whole eye tissue collected at 4-hourly intervals across 24 h from WT and *Per3^–/–^* mice that had been housed in constant darkness, which allows the detection of intrinsic circadian rhythms in the absence of an entraining light/dark cycle. The data show a circadian rhythm in the expression of *Prdm16* in WT mice and a significantly down-regulated expression profile in *Per3^–/–^* mice (Figure 3C; genotype effect *P* < 0.0001). These qPCR data independently demonstrate in an alternative protocol, that *PRDM16* is indeed down-regulated in *Per3^–/–^* compared with WT mice.

### Accumulated Weight Gain in *Per3^–/–^* Mice

**FIGURE 4:**
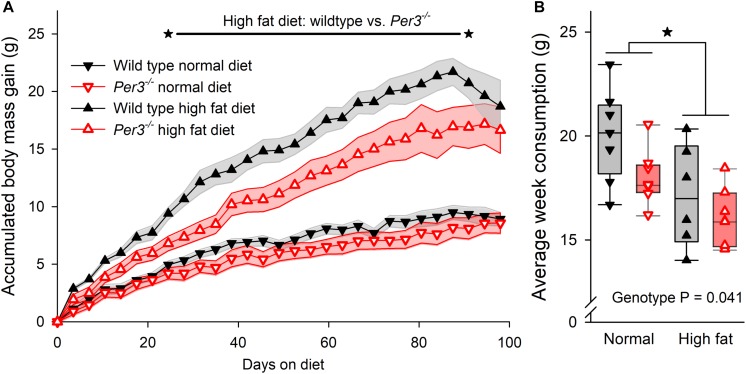
Body mass accumulation in WT and *Per3^–/–^* mice. **(A)** Accumulative body mass gain in wildtype (sold black symbols) and *Per3^–/–^* (open red symbols) mice, fed on normal (downward triangle) and high-fat (upward triangles) diet. The asterisks connected by the black line indicates a significantly (*P* < 0.001–0.05 for *post hoc* multiple comparisons) higher accumulated body mass gain in wildtype mice, compared to *Per3^–/–^* mice on a high fat diet. **(B)** Individual, and boxplot interquartile ranges of average food consumption for both genotypes on both diets, indicating that WT mice eat more food than *Per3^–/–^* mice (*P* < 0.05) irrespective of diet, and that irrespective of genotypes, mice eat less when on a high-fat diet (*P* < 0.05). There were no significant interaction effects. *P < 0.05.

Several clock genes have been associated with metabolic phenotypes in mice (Bass and Takahashi, 2010), and human *PER3* has been linked with BMI and obesity (Lazar et al., 2012; Samblas et al., 2018). Thus, we wanted to investigate further whether down-regulation of *Prdm16* in *Per3^–/–^* mice could affect metabolism and body mass. WT and *Per3^–/–^* mice were fed either a normal or a high fat diet from 4 weeks of age for a period of 14 weeks. Body weight and food consumption were recorded twice per week. During the protocol, mice were housed individually on an LD 12:12 cycle that was advanced by 6 h every 7 days, which represents a chronic jet-lag protocol shown to be stressful in mice (Davidson et al., 2006). Although this is different to the ultradian protocol used for the transcriptome experiment, we chose this approach as being more suitable to identify a metabolic phenotype difference between WT and *Per3^–/–^* mice because exposure to continuously shifted light/dark schedules in mice is associated with metabolic disorders (Kolbe and Oster, 2019). There was a significant effect of genotype, diet and time on body mass accumulation (interaction *P* < 0.0001, *post hoc* multiple comparisons *P* < 0.001–0.05 for WT vs. *Per3^–/–^* mice in high fat diet). Compared to WT, *Per3^–/–^* mice accumulated less body mass when fed on a high fat diet during weeks 4–13 (Figure 4A). Both WT and *Per3^–/–^* mice consumed less food when on the high fat diet compared to the normal diet (Figure 4B), and *Per3^–/–^* mice consumed less than WT on both diets (Figure 4B). This shows that PER3 associates with altered body mass accumulation during early development.

Genes associated with biological pathways are likely to be differentially expressed at varying levels. Thus, for GO pathway level analysis we relaxed the fold change cut-off to include all probes that were significantly differentially expressed between genotypes, as determined by a rank products analysis. This analysis identified 226 probes (103 up-regulated and 123 down regulated in *Per3^–/–^* compared to WT) that mapped to 194 unique genes in whole eye, and 775 probes (299 up regulated and 476 down regulated) that mapped to 672 unique genes in hypothalamus (Supplementary Data File S3). GO enrichment analysis (Webgestalt; Zhang et al., 2005; Liao et al., 2019) for the differentially expressed genes in the eye revealed 14 significant biological processes and molecular functions (FDR < 0.05) including steroid hydroxylase activity, oxidoreductase activity, fatty acid metabolic process, and inflammatory response (Supplementary Data File S4). For the differentially expressed genes in the hypothalamus, there were many more significant GO terms and the top 40 ranked by FDR are shown in Supplementary Data File S5. For the hypothalamus, biological processes and molecular functions included response to oxygen-containing compound, circulatory system development, cell proliferation, double-stranded DNA binding, RNA polymerase II regulatory region DNA binding, tissue development, and transcription regulatory region DNA binding.

**FIGURE 5:**
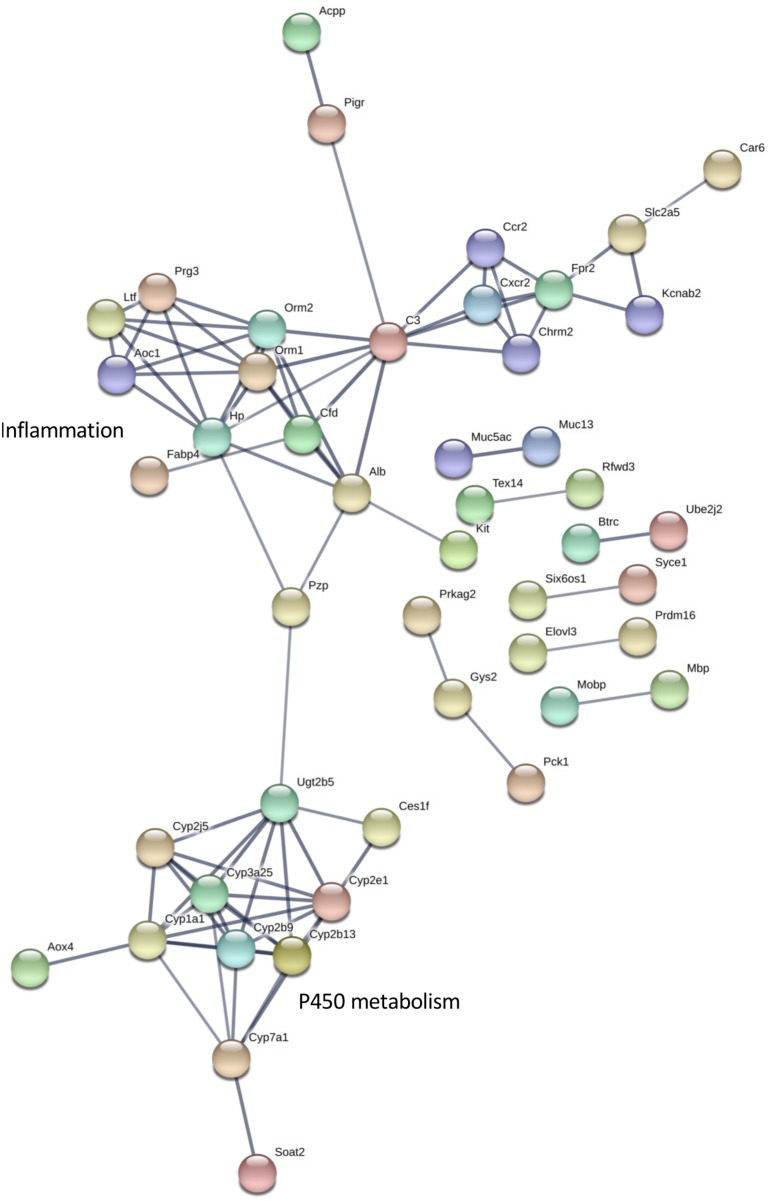
Interaction network for differentially expressed eye transcripts. STRING v11 was used to generate gene/protein interaction networks from transcripts differentially expressed in the eye, according to rank products analysis. The interaction confidence limit was set to high, thickness of edges corresponds to confidence, and unconnected nodes are not displayed. Network clusters associated with inflammation and P450 metabolism are shown. The total number of edges in the network was 85, the expected number derived from the input gene list (*n* = 194) was 36, and the corresponding enrichment *p* value was 1.943e-12.

**FIGURE 6:**
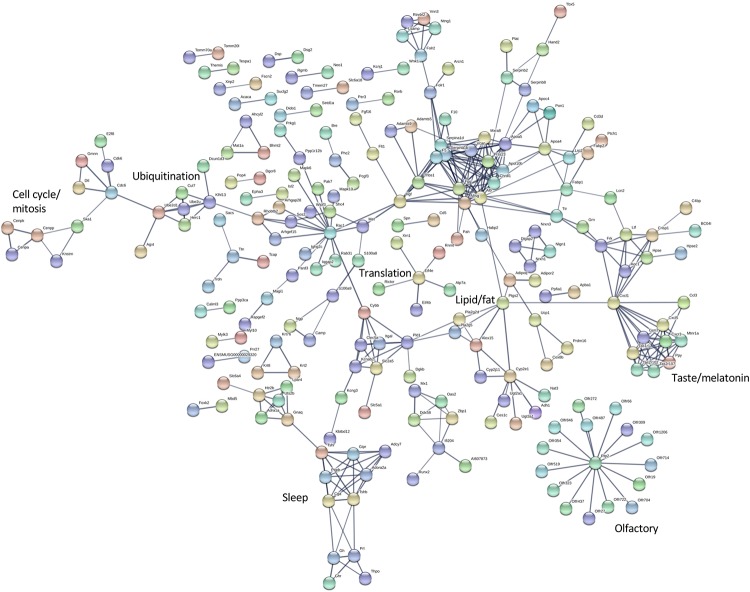
Interaction network for differentially expressed hypothalamus transcripts. STRING v11 was used to generate gene/protein interaction networks from transcripts differentially expressed in the hypothalamus, according to rank products analysis. The interaction confidence limit was set to high, thickness of edges corresponds to confidence, and unconnected nodes are not displayed. Network clusters associated with functions of interest are indicated. The total number of edges in the network was 404, the expected number derived from the input gene list (*n* = 672) was 227, and the corresponding enrichment *p* value was 4.44e-13.

The differentially expressed transcripts identified by rank products analysis also showed significant enrichment for interaction networks (STRING v 11, analysis set for high confidence; whole eye number of edges 85, expected number 36, enrichment *P* = 1.943e-12; hypothalamus number of edges 404, expected number 227, enrichment *P* = 4.44e-13) (Figures 5, 6). The interaction network for the eye contains a sub-network centered around complement component (C3) with related inflammatory functions and another sub-network with several members related to cytochrome P450 metabolism, which were also enriched in several of the terms in the GO analysis (Figure 5). The interaction network for the hypothalamus (Figure 6) contains several clusters of interest that include ubiquitination, cell cycle and mitosis, taste, melatonin, and olfactory receptors. Other clusters have functions that are closely related to sleep including a cluster centered around prostaglandin synthase 2 (*Ptgs2*) involved in lipid/fat metabolism (also containing *Adipoq*, *Adipor2*, *Prdm16*, and *Ucp1*), and an elongated cluster containing prolactin, growth hormone and growth hormone receptor, thyroid stimulating hormone and thyroid stimulating hormone receptor, prostaglandin D2 receptor, and adrenergic and adenosine receptors. The densest cluster in the network contains mainly extracellular matrix proteins with functions related to circulatory activity (Figure 6).

### Expression QTL Correlation Analysis With *Per3* in BXD Mice

**FIGURE 7:**
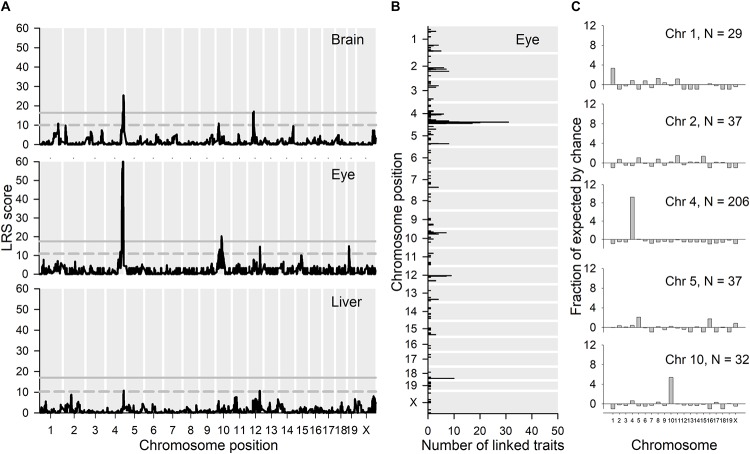
eQTL analyses. **(A)** GeneNetwork was used to map the chromosomal locations of likelihood ratio statistics (LRS scores) for genetic variation linked with *Per3* expression (1421086_at) in brain, eye and liver using interval mapping. **(B)** The top 500 transcripts whose expression correlated with *Per3* expression in eye were extracted and their maximal LRS and number of traits at each location are shown in the figure. **(C)** We then looked at the chromosomes that contain substantial enrichments of maximal expression linkage for the top 500 correlated mRNA transcripts shown in **(B)**, and for each of those chromosomes we looked at which transcripts exhibit these maximal LRS locations. For each of the chromosomes that exhibit substantial maximal linkage, we show the proportional enrichment (fraction of expected, see section “Materials and Methods”) of linked transcripts for each chromosome in **(C)**.

Because of the co-localization with *Per3* of a large proportion of the differentially expressed genes in *Per3^–/–^* mice, we next used expression eQTL database analysis (see text footnote 1) to identify chromosomal loci where genetic variation correlated with *Per3* expression levels in multiple tissue experiments from recombinant backcross strains of C57BL/6J and DBA/2J (BXD) mice. We performed LRS (likelihood ratio statistic; LRS = logarithm of the odds [LOD] × 4.61) score mapping for *Per3* gene expression for brain and peripheral tissue gene expression datasets for BXD mice strains (see text footnote 1; see section “Materials and Methods” for details). Many brain regions showed a highly significant LRS peak for *Per3* expression on distal chromosome 4 at exactly the same region where differentially expressed microarray transcripts co-localized with *Per3* in our data. These brain regions included the nucleus accumbens, hippocampus, eye, cerebellum, prefrontal cortex, and striatum. For convenience, in Figure 7A we show the results of this mapping for whole brain, as well as eye and liver. It should be noted that there is only one dataset available for hypothalamic gene expression, which does not show a significant LRS peak for *Per3* expression on chromosome 4. The liver dataset is characteristic of peripheral tissues that showed a much reduced and often not significant LRS peak at distal chromosome 4 for *Per3* expression. Thus, this linkage appears stronger in brain tissues. The LRS peak at chromosome 4 was particularly significant for the eye (Figure 7A). Although, as expected, this peak centers around *Per3*, it remains significant for a far broader region of distal chromosome 4 that covers many genes (Supplementary Figure S1), including those that we showed to be co-regulated with *Per3* in our transcriptomic analyses (Figure 2).

We next used the BXD gene expression datasets to identify genes whose expression showed a significant correlation with *Per3* expression levels. The top 500 transcripts whose expression covaries with *Per3* in the eye are listed in Supplementary Data File S6. In the LRS map for *Per3* expression in the eye (Figure 7A), in addition to the main LRS peak at chromosome 4, there are also peaks that reach suggestive significance on chromosomes 10, 12, 15, and 19. However, many of the most significantly correlated transcripts from these analyses were also located in the same region at distal chromosome 4 surrounding *Per3* (Figure 7B) and also had maximal LRS expression linkage scores with genetic variation in the same region of chromosome 4 (Figure 7C). Although the majority of the transcripts showed cis eQTL linkage, 19 of the top 100 transcripts had expression linkage at chromosome 4 but were physically located on other chromosomes, and four transcripts were located on chromosome 4 with linkage to other chromosomes. The *P* values for the correlations listed in Supplementary Data File S6 are non-corrected for multiple testing, but even when a recommended three orders of magnitude are subtracted from the *P* values, 96 of the top 100 correlations remain significant [see Supplementary Figure S2a for example of gene expression scatter plot for the highest ranked correlation apart from *Per3* transcripts (*Vamp3*)].

In our transcriptomics data, *Prdm16* was the most down-regulated transcript in both hypothalamic and eye tissue in *Per3^–/–^* mice. Several *Prdm16* transcripts appear in the eye *Per3* gene expression correlation list (Supplementary Data File S6). The *Prdm16* transcript with the highest-ranking *p* value has a strong positive correlation with *Per3* expression (Supplementary Figure S2b). While this agrees with our own transcriptome data (i.e., low levels of *Per3* expression are associated with low levels of *Prdm16* expression), it should be noted that other *Prdm16* transcripts have negative correlations with *Per3* expression.

The list of the top 500 transcripts whose expression covaried with *Per3* in the eye was submitted to Webgestalt for GO analysis. This identified 322 unique coding genes which were enriched for many significant GO terms for biological processes and molecular functions that included kinase binding, ribonucleotide binding, response to light stimulus, positive regulation of gene expression, regulation of myelination, ubiquitin protein ligase binding, and regulation of cell cycle (see Supplementary Data File S7 for top 40). Several of these terms show overlap with the node clusters for the interaction network for differentially expressed genes in the hypothalamus, e.g., cell cycle, ubiquitination, translation (Figure 6). It should also be noted that the interaction network for differentially expressed genes in the eye contained two connected nodes for *Mobp* (myelin associated oligodendrocyte basic protein) and *Mbp* (myelin basic protein) (Figure 4). Although these two specific genes do not feature in the top 500 covarying genes from the BXD datasets, it nevertheless points to a potential overlap in the regulation of molecular function.

## Discussion

In this study, we replicated the reduced negative masking behavioral phenotype that we had previously observed in the *Per3^–/–^* mice subjected to an ultradian light:dark cycle (van der Veen and Archer, 2010). We chose to use this behavioral difference between WT and *Per3^–/–^* mice to investigate the differential gene expression associated with the absence of a functional PER3 protein in eye and hypothalamic tissue within a light/dark protocol that we knew produced marked differential behavioral responses between WT and *Per3^–/–^* mice. However, we acknowledge that we cannot distinguish between differential gene expression induced by this protocol and any underlying differences in gene expression that may not be related to the protocol, which would have required a transcriptomic comparison at baseline LD 12:12 conditions. Nevertheless, we chose the protocol to maximize the opportunity to detect differential gene expression between the genotypes, which is what we report here.

Analyses of differentially expressed transcripts identified genes whose function were not related directly with circadian rhythms but more generally with functions such as transcription/translation, ubiquitination, cell cycle, and metabolism. More specific functions were related to olfaction, taste, lipid/fat regulation, ion channels and hormones and hormone receptors. Some of these have been implicated with sleep regulation and homeostasis and this agrees with our previous findings for differential expression in humanized mice expressing either the 4- or 5-repeat alleles for the human *PER3* VNTR polymorphism. Genes of particular interest include those for prostaglandin endoperoxide synthase 2 (*Ptgs2*) and prostaglandin D2 receptor (*Ptgdr*), which has been linked with NREM sleep regulation (Oishi et al., 2015). *Ptgs2* is also expressed together with the brain specific sleep-loss correlate *Homer1a* as part of the recovery mechanism from glutamate-induced neuronal hyperactivity (Maret et al., 2007). Polymorphisms in the adenosine receptor gene (*Adora2a*) affect sleep homeostasis (Retey et al., 2007) and interact with the *PER3* VNTR polymorphism to determine cognitive performance during sleep restriction (Rupp et al., 2013). Growth hormone (*Gh*, *Ghr*), prolactin (*Prl*), and thyroid stimulating hormone (*Tshb*, *Tshr*) are all associated with sleep/wake regulation, e.g., prolactin and REM sleep (Machado et al., 2017), thyroid stimulating hormone and sleep deprivation (Pereira and Andersen, 2014), and relations between age-related growth hormone secretion and REM sleep (Van Cauter et al., 2000). The melatonin receptor *Mtnr1a* is differentially expressed in the hypothalamus and has links with circadian rhythms and also with exhaustion in shift workers (Sulkava et al., 2017), insomnia in schizophrenics (Park et al., 2011), and with Alzheimer’s (Sulkava et al., 2018). The human *PER3* VNTR polymorphism has been linked with myelination and brain structure (Bollettini et al., 2017; Dewandre et al., 2018), and it is interesting that two genes associated with myelination (*Mobp*, *Mbp*) were differentially expressed in the eye.

We showed that the most down-regulated gene in both eye and hypothalamus in the *Per3^–/–^* mice was *Prdm16* and, with qPCR, we observed reduced circadian rhythmicity of *PRDM16* in conditions of constant darkness in the eye. *Prdm16* is located close to *Per3* on distal chromosome 4 and showed highly correlated levels of expression with *Per3* in BXD lines. PRDM16 is a zinc finger transcription factor that regulates a range of developmental processes that include the neocortex (Inoue et al., 2017), cardiovascular system and hematopoiesis (Chi and Cohen, 2016), and brown fat development (Seale et al., 2007). PRDM16 influences brown fat development by regulating expression of uncoupling protein 1, *Ucp1* (Iida et al., 2015), which was also differentially expressed in the hypothalamus. PRDM16 enhances *Ucp1* gene expression via a direct interaction with a subunit (MED1) of the mediator component of the RNA polymerase II complex (Iida et al., 2015). This regulation by PRDM16 also involves interaction with thyroid hormones receptors at thyroid receptor elements in gene promoters (Iida et al., 2015), which is perhaps also relevant for the differential expression of *Tshb* and *Tshr* that we observed in our data. It has also been shown that binding of PRDM16 to MED1 alters chromatin structure and regulates the activity of super-enhancers that drive expression of co-regulated genes (Harms et al., 2014). Because PRDM16 was consistently down-regulated in both tissues, which was confirmed with qPCR and agreed with the BXD expression correlation data, we investigated if there was a body mass phenotype in the *Per3^–/–^* mice. We indeed found that *Per3^–/–^* mice accumulated less body mass than WT mice when fed on a high-fat diet from age 4 weeks. Previously it has been shown that *Per3^–/–^* mice (albeit on a different background and with different lighting conditions) accumulated more body mass than WT when fed a high-fat diet (Dallmann and Weaver, 2010). However, that was only apparent from age 20 weeks and the reduced body mass accumulation that we found in the *Per3^–/–^* mice agrees with data from younger mice in the previous study. Indeed, after 13 weeks in our study the difference in accumulated body mass gain is no longer significant and it is possible that with extended measurements we would have seen an increase in body mass accumulation in the *Per3^–/–^* mice compared to WT mice, as was observed in older mice in the previous study. Thus, the influence of PER3/PRDM16 on body mass accumulation seems to be age-dependent, but may also be strain dependent as has been noted for metabolic phenotypes in other clock gene knock outs (Johnston et al., 2009). It is also interesting to note that both our study and the previous one by Dallmann and Weaver observed reduced food intake in both genotypes in the high fat diet compared to normal diet, and also reduced food intake in *Per3^–/–^* mice compared to WT.

We found that there was a highly significant LRS linkage peak between *Per3* expression and genetic variation at distal chromosome 4 and that the LRS peak width corresponded almost exactly with the region where we identified differentially expressed transcripts in the *Per3^–/–^* mice that colocalized around *Per3*. The only other core circadian clock gene to show a significant LRS peak for the BXD eye RNA dataset was *Nr1d1* (*Reverb*α) but the LRS peak was located on chromosome 4 while *Nr1d1* itself is on chromosome 11. There was a suggestive LRS peak for *Clock* at chromosome 1 that coincided exactly with the locations of *Per2* and *Hdac4*. Apart from *Per3*, none of the other circadian clock genes were found to covary in expression with other genes located in the same chromosomal region. However, some of the other core clock genes did show corelated expression with other known clock elements, whereas *Per3* did not. For example, the gene with expression most highly correlated with that of *Arntl* was *Clock*, and the fourth and seventh highest correlations were with *Per2* and *Dbp*, respectively. Similarly, for *Per2* the second and third highest expression correlations were with *Tef* and *Dbp*, respectively. Thus, unlike *Per3*, the expression of other clock genes is closely linked with the expression of other clock elements and not neighboring non-circadian genes. These observations are consistent with what we know about *Per3* – it is a non-essential clock gene with many associated non-circadian phenotypes.

Previously, we created knock in mice homozygous for either the 4- or 5-repeat allele of the primate-specific human *PER3* VNTR polymorphism (Hasan et al., 2014). We investigated differential gene expression in the hypothalamus in those mice after 12 h of sleep deprivation. We compared the lists of significantly differentially expressed genes from this and the previous experiment. From the Venn diagram in Supplementary Figure S3 (see also Supplementary Table S2 for lists of overlapping genes), we see that there is overlap between the lists for the KO eye and hypothalamus from this study but less overlap between those lists and the genes that were differentially expressed between the 4- and 5-repeat homozygous mice for the hypothalamus. It is also evident that there is little overlap between the KO eye and hypothalamus and the genes that covary with *Per3* in the eye in the BXD mice, although there is more overlap between those BXD genes and the differentially expressed VNTR alleles. From this we can speculate that when *Per3* is present in BXD mice, there is a set of genes colocalized at distal chromosome 4 whose expression is coregulated together with *Per3*. The absence of PER3 protein only directly affects the expression of a smaller number of genes in this region. Expression of *Per3* with either the 4- or 5-repeat alleles affects the expression of a larger number of coregulated genes but again only a small subset of the BXD covarying genes. This implies that PER3 itself only directly influences a small subset of other genes in this coregulated chromosomal region.

All of the data that we have presented here are consistent with the hypothesis that much of the gene expression in the distal region of mouse chromosome 4 is co-regulated. Such chromosomal regions of transcriptional co-regulation have been shown to be related directly with chromatin architecture whereby chromosomal regions of closely interacting chromatin form distinguishable units called TADs. Control elements within TADs can enhance gene expression within the TAD and suppress gene expression in a neighboring TAD (Dixon et al., 2016). In mammals, neighboring TADs are often co-regulated in a super structure whereby connecting chromatin loops produce strong chromatin interaction between TADs, with neighboring TADs in either active or inactive expression states (Szabo et al., 2019). To investigate the possibility that distal chromosome 4 contains a co-regulated TAD neighborhood, we used a Hi-C browser (see section “Materials and Methods”) to show chromatin interaction and associated predicted TADs in a published mouse cortex Hi-C dataset (Dixon et al., 2012). The results confirm that there is indeed an island of three interacting TADs at distal mouse chromosome 4 that corresponds almost exactly with the region (149–157 Mb) where we identified a cluster of differentially expressed transcripts in mouse eye and hypothalamus in our data (Supplementary Figure S4). This also corresponds precisely with the position and width of the eQTL LRS peak for *Per3* expression, and with the chromosomal position of genes whose expression in BXD lines significantly covaries with *Per3* expression.

Topologically associating domain boundaries are defined by specific chromatin features including binding of protein elements such as CCCTC-binding factor (CTCF) and cohesion complex (Dixon et al., 2016). From the heatmap in Supplementary Figure S4b, it can be seen that *Per3* is located very close to the boundary between two predicted neighboring TADs. The *Per3* functional knock out mouse was created using a PGK-NEO cassette that replaced exon 3 and the intron between exon 3 and exon 4, introducing a stop codon in all three reading frames, resulting in undetectable levels of PER3 protein (Shearman et al., 2000). However, addition of the cassette increased the detectable size of the *Per3* mRNA by 2 kb and expression levels of *Per3* mRNA were significantly reduced in the suprachiasmatic nucleus and skeletal muscle, which is in line with our reduced expression for *Per3* in the hypothalamus of *Per3^–/–^* mice. Thus, we cannot exclude the possibility that insertion of an extra 2 kb in the *Per3* KO transgene has somehow altered chromatin architecture in this region and affected the TAD boundary dynamics, leading to altered regulation of local gene expression, including that of *Per3* itself.

A similar TAD island is also present at the region of human chromosome 1 that is syntenic with mouse distal chromosome 4, where *PER3* is also positioned at the boundary between two neighboring TADs. The human *PER3* VNTR polymorphism is an insertion of only 54 bp in exon 19 (in the current human genome assembly GRCh38.p13) and rather than altering chromatin structure and TAD dynamics, it seems more likely that this coding-region polymorphism affects PER3 protein function which leads to the observed phenotypic associations (although the VNTR does overlap with a regulatory open chromatin mark). However, the human genome assembly also shows that the *PER3* gene overlaps with a long non-coding RNA (lncRNA) transcript (lncZ98884.1-201) present on the opposite strand. The transcriptional start site of the lncRNA coincides with *PER3* exon 18, where there is also an H3K27Ac histone acetylation mark and a DNaseI sensitivity site, which are both markers of chromatin modification and accessibility associated with regulatory elements at gene transcription start sites. Thus, transcription of the lncRNA could be affected by the adjacent *PER3* VNTR. The function of this particular lncRNA is unknown, but lncRNAs mainly regulate transcription and translation and it is possible that this antisense transcript regulates *PER3* expression in addition to other genes. The current mouse genome annotation does not include an antisense lncRNA within *Per3* but this cannot be ruled out.

Some developmental diseases caused by chromosome abnormalities have been shown to disrupt TAD architecture that regulates developmental gene expression (e.g., F syndrome caused by an inversion on chromosome 2; see Lupianez et al., 2016, for review). Human 1p36 deletion syndrome is caused by a chromosomal deletion in a region of chromosome 1 that is syntentic with mouse distal chromosome 4. Developmental abnormalities include intellectual disability, brain anomalies, vision problems, hearing loss, facial anomalies, and heart defects (Jordan et al., 2015). Disruption to several genes has been associated with the syndrome, including *PRDM16* and *KCNAB2*, which were differentially expressed in the present study. Thus, we hypothesize that 1p36 deletion disrupts the conserved TAD architecture in this region.

We and others have previously shown that PER3 associates with a wide range of non-circadian phenotypes. In this study, we have demonstrated that the functional knock out of *Per3* led to changes in the expression of genes with functions related to those phenotypes. A significant number of the co-regulated genes also occur in close proximity to *Per3* on distal chromosome 4, where eQTL analyses show there is a significant linkage peak that covers the same chromosomal location and many genes in that region show correlated expression with *Per3* in BXD lines. Chromatin interaction analyses showed that this region is a TAD superdomain which explains the local co-regulation that we observed in both our data and the eQTL data. Thus, expression of *Per3* (and human *PER3* at syntenic chromosome 1) is co-regulated with a number of neighboring genes which presumably explains its association with a range of pleiotropic phenotypes. The links between these phenotypes and human disease underlines the relevance for a greater awareness of genetic variation and regulation in this chromosomal region.

## Data Availability Statement

The datasets for this study can be found in the GEO database, under accession number GSE143650: https://www.ncbi.nlm.nih.gov/geo/query/acc.cgi?acc=GSE143650.

## Ethics Statement

All experimental procedures were approved by the University of Surrey, Animal Ethics Committee and carried out under a United Kingdom Home Office License.

## Author Contributions

DVdV, JJ, D-JD, and SA conceived the research and wrote the manuscript. DVdV, S-EB, and SA performed the research. DVdV, EL, and SA analyzed the data.

## Conflict of Interest

The authors declare that the research was conducted in the absence of any commercial or financial relationships that could be construed as a potential conflict of interest.

## Footnotes

1 genenetwork.org (accessed June 2019).

2 promoter.bx.psu.edu/hi-c

## References

[B1] Alexander M. Burch J. B. Steck S. E. Chen C. F. Hurley T. G. Cavicchia P. (2015). Case-control study of the PERIOD3 clock gene length polymorphism and colorectal adenoma formation. *Oncol. Rep.* 33 935–941. 10.3892/or.2014.3667 25501848 PMC4306271

[B2] Archer S. N. Oster H. (2015). How sleep and wakefulness influence circadian rhythmicity: effects of insufficient and mistimed sleep on the animal and human transcriptome. *J. Sleep Res.* 24 476–493. 10.1111/jsr.12307 26059855

[B3] Archer S. N. Robilliard D. L. Skene D. J. Smits M. Williams A. Arendt J. (2003). A length polymorphism in the circadian clock gene Per3 is linked to delayed sleep phase syndrome and extreme diurnal preference. *Sleep* 26 413–415. 10.1093/sleep/26.4.413 12841365

[B4] Bae K. Jin X. Maywood E. S. Hastings M. H. Reppert S. M. Weaver D. R. (2001). Differential functions of *mPer1*, *mPer2*, and *mPer3* in the SCN circadian clock. *Neuron* 30 525–536. 10.1016/s0896-6273(01)00302-6 11395012

[B5] Baizabal J. M. Mistry M. Garcia M. T. Gomez N. Olukoya O. Tran D. (2018). The epigenetic state of PRDM16-regulated enhancers in radial glia controls cortical neuron position. *Neuron* 99 239–241. 10.1016/j.neuron.2018.06.031 30001508

[B6] Bass J. Takahashi J. S. (2010). Circadian integration of metabolism and energetics. *Science* 330 1349–1354. 10.1126/science.1195027 21127246 PMC3756146

[B7] Benedetti F. Dallaspezia S. Colombo C. Pirovano A. Marino E. Smeraldi E. (2008). A length polymorphism in the circadian clock gene Per3 influences age at onset of bipolar disorder. *Neurosci. Lett.* 445 184–187. 10.1016/j.neulet.2008.09.002 18789374

[B8] Bennett B. J. Farber C. R. Orozco L. Kang H. M. Ghazalpour A. Siemers N. (2010). A high-resolution association mapping panel for the dissection of complex traits in mice. *Genome Res.* 20 281–290. 10.1101/gr.099234.109 20054062 PMC2813484

[B9] Bollettini I. Melloni E. M. Aggio V. Poletti S. Lorenzi C. Pirovano A. (2017). Clock genes associate with white matter integrity in depressed bipolar patients. *Chronobiol. Int.* 34 212–224. 10.1080/07420528.2016.1260026 27996307

[B10] Chellappa S. L. Viola A. U. Schmidt C. Bachmann V. Gabel V. Maire M. (2012). Human melatonin and alerting response to blue-enriched light depend on a polymorphism in the clock gene *PER3*. *J. Clin. Endocrinol. Metab.* 97 E433–E437. 10.1210/jc.2011-2391 22188742

[B11] Chi J. Cohen P. (2016). The multifaceted roles of PRDM16: adipose biology and beyond. *Trends Endocrinol. Metab.* 27 11–23. 10.1016/j.tem.2015.11.005 26688472

[B12] Dallmann R. Weaver D. R. (2010). Altered body mass regulation in male mPeriod mutant mice on high-fat diet. *Chronobiol. Int.* 27 1317–1328. 10.3109/07420528.2010.489166 20653457 PMC2911971

[B13] Davidson A. J. Sellix M. T. Daniel J. Yamazaki S. Menaker M. Block G. D. (2006). Chronic jet-lag increases mortality in aged mice. *Curr. Biol.* 16 R914–R916. 17084685 10.1016/j.cub.2006.09.058 PMC1635966

[B14] Dewandre D. Atienza M. Sanchez-Espinosa M. P. Cantero J. L. (2018). Effects of PER3 clock gene polymorphisms on aging-related changes of the cerebral cortex. *Brain Struct. Funct.* 223 597–607. 10.1007/s00429-017-1513-0 28900721

[B15] Dixon J. R. Gorkin D. U. Ren B. (2016). Chromatin domains: the unit of chromosome organization. *Mol. Cell* 62 668–680. 10.1016/j.molcel.2016.05.018 27259200 PMC5371509

[B16] Dixon J. R. Selvaraj S. Yue F. Kim A. Li Y. Shen Y. (2012). Topological domains in mammalian genomes identified by analysis of chromatin interactions. *Nature* 485 376–380. 10.1038/nature11082 22495300 PMC3356448

[B17] Groeger J. A. Viola A. U. Lo J. C. von Schantz M. Archer S. N. Dijk D. J. (2008). Early morning executive functioning during sleep deprivation is compromised by a PERIOD3 polymorphism. *Sleep* 31 1159–1167. 18714788 PMC2542962

[B18] Guess J. Burch J. B. Ogoussan K. Armstead C. A. Zhang H. Wagner S. (2009). Circadian disruption, *Per3*, and human cytokine secretion. *Integr. Cancer Ther.* 8 329–336. 10.1177/1534735409352029 19926609 PMC2959170

[B19] Harms M. J. Ishibashi J. Wang W. Lim H. W. Goyama S. Sato T. (2014). Prdm16 is required for the maintenance of brown adipocyte identity and function in adult mice. *Cell Metab.* 19 593–604. 10.1016/j.cmet.2014.03.007 24703692 PMC4012340

[B20] Hasan S. van der Veen D. R. Winsky-Sommerer R. Dijk D. J. Archer S. N. (2011). Altered sleep and behavioral activity phenotypes in PER3-deficient mice. *Am. J. Physiol. Regul. Integr. Comp. Physiol.* 301 R1821–R1830. 10.1152/ajpregu.00260.2011 21957163

[B21] Hasan S. van der Veen D. R. Winsky-Sommerer R. Hogben A. Laing E. E. Koentgen F. (2014). A human sleep homeostasis phenotype in mice expressing a primate-specific *PER3* variable-number tandem-repeat coding-region polymorphism. *FASEB J.* 28 2441–2454. 10.1096/fj.13-240135 24577121 PMC4046067

[B22] Iida S. Chen W. Nakadai T. Ohkuma Y. Roeder R. G. (2015). PRDM16 enhances nuclear receptor-dependent transcription of the brown fat-specific Ucp1 gene through interactions with Mediator subunit MED1. *Genes Dev.* 29 308–321. 10.1101/gad.252809.114 25644605 PMC4318147

[B23] Inoue M. Iwai R. Tabata H. Konno D. Komabayashi-Suzuki M. Watanabe C. (2017). Prdm16 is crucial for progression of the multipolar phase during neural differentiation of the developing neocortex. *Development* 144 385–399. 10.1242/dev.136382 27993981

[B24] Johnston J. D. Frost G. Otway D. T. (2009). Adipose tissue, adipocytes and the circadian timing system. *Obes. Rev.* 10(Suppl. 2), 52–60. 10.1111/j.1467-789x.2009.00665.x 19849802

[B25] Jordan V. K. Zaveri H. P. Scott D. A. (2015). 1p36 deletion syndrome: an update. *Appl. Clin. Genet.* 8 189–200. 10.2147/TACG.S65698 26345236 PMC4555966

[B26] Karthikeyan R. Marimuthu G. Ramasubramanian C. Arunachal G. BaHammam A. S. Spence D. W. (2014a). Association of *Per3* length polymorphism with bipolar I disorder and schizophrenia. *Neuropsychiatr. Dis. Treat.* 10 2325–2330. 10.2147/NDT.S73765 25525361 PMC4267513

[B27] Karthikeyan R. Marimuthu G. Sooriyakumar M. BaHammam A. S. Spence D. W. Pandi-Perumal S. R. (2014b). *Per3* length polymorphism in patients with type 2 diabetes mellitus. *Horm. Mol. Biol. Clin. Investig.* 18 145–149. 10.1515/hmbci-2013-0049 25390010

[B28] Kolbe I. Oster H. (2019). Chronodisruption, metabolic homeostasis, and the regulation of inflammation in adipose tissues. *Yale J. Biol. Med.* 92 317–325. 31249492 PMC6585521

[B29] Laing E. Smith C. P. (2010). RankProdIt: a web-interactive Rank Products analysis tool. *BMC Res. Notes* 3:221. 10.1186/1756-0500-3-221 20691047 PMC2930644

[B30] Lazar A. S. Slak A. Lo J. C. Santhi N. von Schantz M. Archer S. N. (2012). Sleep, diurnal preference, health, and psychological well-being: a prospective single-allelic-variation study. *Chronobiol. Int.* 29 131–146. 10.3109/07420528.2011.641193 22324552

[B31] Liao Y. Wang J. Jaehnig E. J. Shi Z. Zhang B. (2019). WebGestalt 2019: gene set analysis toolkit with revamped UIs and APIs. *Nucleic Acids Res.* 47 W199–W205. 10.1093/nar/gkz401 31114916 PMC6602449

[B32] Lo J. C. Groeger J. A. Santhi N. Arbon E. L. Lazar A. S. Hasan S. (2012). Effects of partial and acute total sleep deprivation on performance across cognitive domains, individuals and circadian phase. *PLoS One* 7:e45987. 10.1371/journal.pone.0045987 23029352 PMC3454374

[B33] Lupianez D. G. Spielmann M. Mundlos S. (2016). Breaking TADs: how alterations of chromatin domains result in disease. *Trends Genet.* 32 225–237. 10.1016/j.tig.2016.01.003 26862051

[B34] Machado R. B. Rocha M. R. Suchecki D. (2017). Brain prolactin is involved in stress-induced REM sleep rebound. *Horm. Behav.* 89 38–47. 10.1016/j.yhbeh.2016.12.004 28017595

[B35] Maret S. Dorsaz S. Gurcel L. Pradervand S. Petit B. Pfister C. (2007). Homer1a is a core brain molecular correlate of sleep loss. *Proc. Natl. Acad. Sci. U.S.A.* 104 20090–20095. 10.1073/pnas.0710131104 18077435 PMC2148427

[B36] Mulligan M. K. Mozhui K. Prins P. Williams R. W. (2017). GeneNetwork: a toolbox for systems genetics. *Methods Mol. Biol.* 1488 75–120. 10.1007/978-1-4939-6427-7_4 27933521 PMC7495243

[B37] Oishi Y. Yoshida K. Scammell T. E. Urade Y. Lazarus M. Saper C. B. (2015). The roles of prostaglandin E2 and D2 in lipopolysaccharide-mediated changes in sleep. *Brain Behav. Immun.* 47 172–177. 10.1016/j.bbi.2014.11.019 25532785 PMC4468012

[B38] Park H. J. Park J. K. Kim S. K. Cho A. R. Kim J. W. Yim S. V. (2011). Association of polymorphism in the promoter of the melatonin receptor 1A gene with schizophrenia and with insomnia symptoms in schizophrenia patients. *J. Mol. Neurosci.* 45 304–308. 10.1007/s12031-011-9522-6 21526376

[B39] Paxinos G. Franklin K. B. J. (2001). *The Mouse Brain in Stereotaxic Coordinates.* Cambridge, MA: Academic Press.

[B40] Pendergast J. S. Niswender K. D. Yamazaki S. (2012). Tissue-specific function of Period3 in circadian rhythmicity. *PLoS One* 7:e30254. 10.1371/journal.pone.0030254 22253927 PMC3256228

[B41] Pereira J. C. Jr. Andersen M. L. (2014). The role of thyroid hormone in sleep deprivation. *Med. Hypotheses* 82 350–355. 10.1016/j.mehy.2014.01.003 24468575

[B42] Ramanathan C. Xu H. Khan S. K. Shen Y. Gitis P. J. Welsh D. K. (2014). Cell type-specific functions of period genes revealed by novel adipocyte and hepatocyte circadian clock models. *PLoS Genet.* 10:e1004244. 10.1371/journal.pgen.1004244 24699442 PMC3974647

[B43] Retey J. V. Adam M. Khatami R. Luhmann U. F. Jung H. H. Berger W. (2007). A genetic variation in the adenosine A2A receptor gene (ADORA2A) contributes to individual sensitivity to caffeine effects on sleep. *Clin. Pharmacol. Ther.* 81 692–698. 10.1038/sj.clpt.6100102 17329997

[B44] Rupp T. L. Wesensten N. J. Newman R. Balkin T. J. (2013). *PER3* and *ADORA2A* polymorphisms impact neurobehavioral performance during sleep restriction. *J. Sleep Res.* 22 160–165. 10.1111/j.1365-2869.2012.01062.x 23171222

[B45] Saba L. Bhave S. V. Grahame N. Bice P. Lapadat R. Belknap J. (2006). Candidate genes and their regulatory elements: alcohol preference and tolerance. *Mamm. Genome* 17 669–688. 10.1007/s00335-005-0190-0 16783646

[B46] Samblas M. Milagro F. I. Mansego M. L. Marti A. Martinez J. A. (2018). *PTPRS* and *PER3* methylation levels are associated with childhood obesity: results from a genome-wide methylation analysis. *Pediatr. Obes.* 13 149–158. 10.1111/ijpo.12224 28614626

[B47] Seale P. Kajimura S. Yang W. Chin S. Rohas L. M. Uldry M. (2007). Transcriptional control of brown fat determination by PRDM16. *Cell Metab.* 6 38–54. 10.1016/j.cmet.2007.06.001 17618855 PMC2564846

[B48] Shearman L. P. Jin X. Lee C. Reppert S. M. Weaver D. R. (2000). Targeted disruption of the *mPer3* gene: subtle effects on circadian clock function. *Mol. Cell. Biol.* 20 6269–6275. 10.1128/mcb.20.17.6269-6275.2000 10938103 PMC86101

[B49] Sulkava S. Muggalla P. Sulkava R. Ollila H. M. Peuralinna T. Myllykangas L. (2018). Melatonin receptor type 1A gene linked to Alzheimer’s disease in old age. *Sleep* 41:zsy103. 10.1093/sleep/zsy103 29982836 PMC6047434

[B50] Sulkava S. Ollila H. M. Alasaari J. Puttonen S. Harma M. Viitasalo K. (2017). Common genetic variation near melatonin receptor 1A gene linked to job-related exhaustion in shift workers. *Sleep* 40:zsw011. 10.1093/sleep/zsw011 28364478 PMC5806557

[B51] Szabo Q. Bantignies F. Cavalli G. (2019). Principles of genome folding into topologically associating domains. *Sci. Adv.* 5:eaaw1668. 10.1126/sciadv.aaw1668 30989119 PMC6457944

[B52] Van Cauter E. Leproult R. Plat L. (2000). Age-related changes in slow wave sleep and REM sleep and relationship with growth hormone and cortisol levels in healthy men. *JAMA* 284 861–868. 10938176 10.1001/jama.284.7.861

[B53] van der Veen D. R. Archer S. N. (2010). Light-dependent behavioral phenotypes in PER3-deficient mice. *J. Biol. Rhythms* 25 3–8. 10.1177/0748730409356680 20075295

[B54] Vandewalle G. Archer S. N. Wuillaume C. Balteau E. Degueldre C. Luxen A. (2009). Functional magnetic resonance imaging-assessed brain responses during an executive task depend on interaction of sleep homeostasis, circadian phase, and *PER3* genotype. *J. Neurosci.* 29 7948–7956. 10.1523/JNEUROSCI.0229-09.2009 19553435 PMC6666044

[B55] Vandewalle G. Archer S. N. Wuillaume C. Balteau E. Degueldre C. Luxen A. (2011). Effects of light on cognitive brain responses depend on circadian phase and sleep homeostasis. *J. Biol. Rhythms* 26 249–259. 10.1177/0748730411401736 21628552

[B56] Viola A. U. Archer S. N. James L. M. Groeger J. A. Lo J. C. Skene D. J. (2007). *PER3* polymorphism predicts sleep structure and waking performance. *Curr. Biol.* 17 613–618. 10.1016/j.cub.2007.01.073 17346965

[B57] Viola A. U. Chellappa S. L. Archer S. N. Pugin F. Gotz T. Dijk D. J. (2012). Interindividual differences in circadian rhythmicity and sleep homeostasis in older people: effect of a *PER3* polymorphism. *Neurobiol. Aging* 33 1010.e17–1010.e27. 10.1016/j.neurobiolaging.2011.10.024 22169200

[B58] Wirth M. Burch J. Violanti J. Burchfiel C. Fekedulegn D. Andrew M. (2013). Association of the Period3 clock gene length polymorphism with salivary cortisol secretion among police officers. *Neuro Endocrinol. Lett.* 34 27–37. 23524621 PMC3655703

[B59] Xiao Y. Hsiao T. H. Suresh U. Chen H. I. Wu X. Wolf S. E. (2014). A novel significance score for gene selection and ranking. *Bioinformatics* 30 801–807. 10.1093/bioinformatics/btr671 22321699 PMC3957066

[B60] Zeng H. C. Bae Y. Dawson B. C. Chen Y. Bertin T. Munivez E. (2017). MicroRNA miR-23a cluster promotes osteocyte differentiation by regulating TGF-beta signalling in osteoblasts. *Nat. Commun.* 8:15000. 10.1038/ncomms15000 28397831 PMC5394267

[B61] Zhang B. Kirov S. Snoddy J. (2005). WebGestalt: an integrated system for exploring gene sets in various biological contexts. *Nucleic Acids Res.* 33 W741–W748. 15980575 10.1093/nar/gki475 PMC1160236

[B62] Zou Y. Liao G. Liu Y. Wang Y. Yang Z. Lin Y. (2008). Association of the 54-nucleotide repeat polymorphism of *hPer3* with heroin dependence in Han Chinese population. *Genes Brain Behav.* 7 26–30. 17451453 10.1111/j.1601-183X.2007.00314.x

