# Supplementary material for: A Topological Cluster of Differentially Regulated Genes in Mice Lacking PER3
Source: Front Mol Neurosci. 2020 Feb 13;13:15. doi: 10.3389/fnmol.2020.00015 (PMC7031657; doi:10.3389/fnmol.2020.00015)
Supplement: Supplementary file 8 [file Data_Sheet_1.PDF]

Supplementary Material

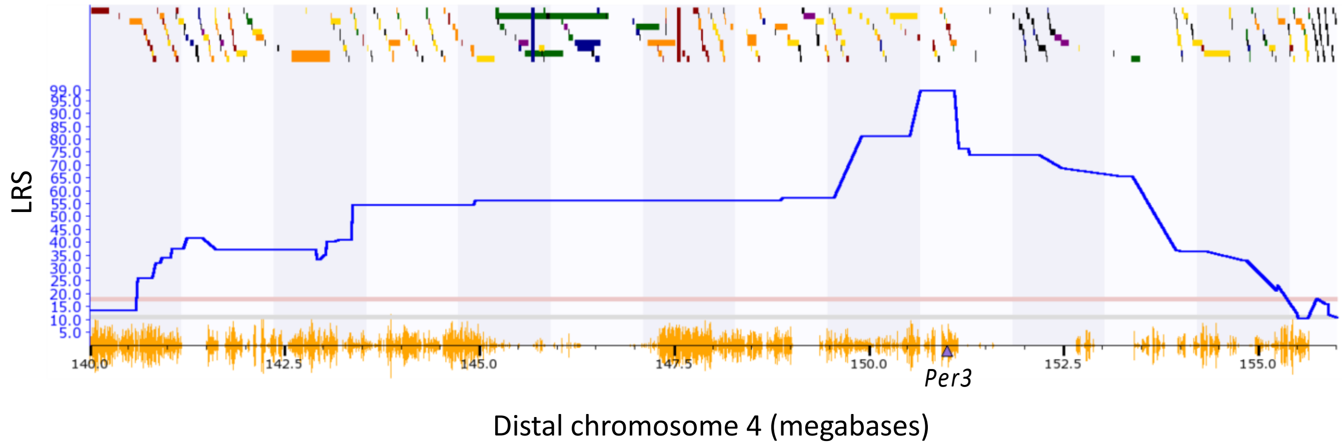

Figure S1. Likelihood ratio statistics (LRS) score (blue line) for genetic variation linked with *Per3* expression (1421086_at) in the eye for distal chromosome 4 (140.0 – 156.0 Mb). The red horizontal bar represents statistical significance for LRS and the grey bar represents suggestive significance levels. Triangle on the x axis indicates the chromosomal position of *Per3*. The density of genetic variation across the region is shown by orange bars on the x axis. The positions of mapped genes/transcripts in the region are shown by coloured bars at the top of the figure.

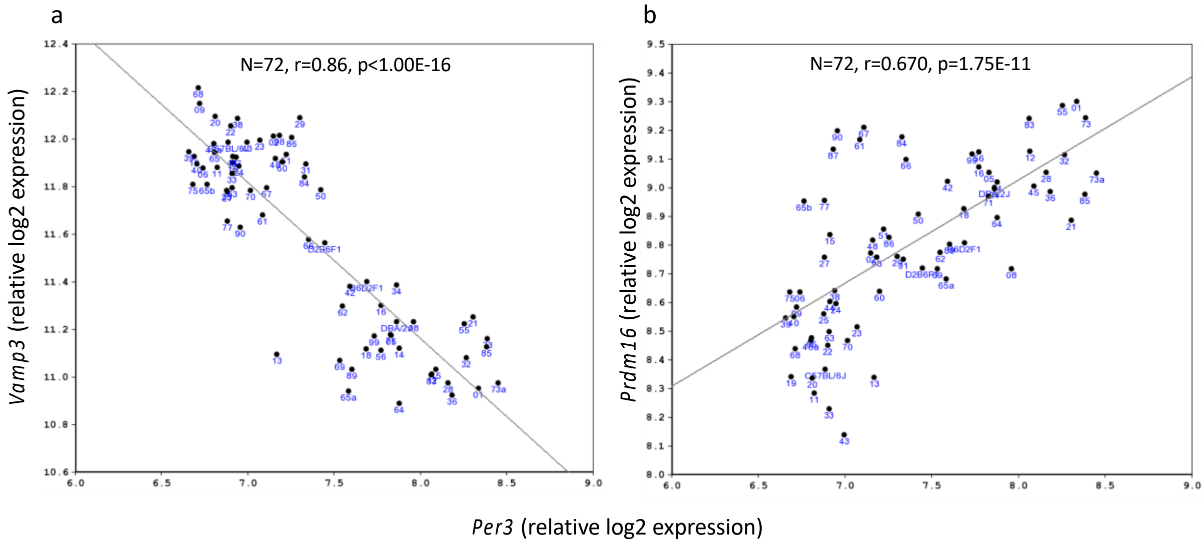

Figure S2. BXD gene expression highly correlated with *Per3*. (a) Expression levels of *Vamp3* showed the highest correlation with expression levels for *Per3* in BXD strains (r=0.862, p<1.00e-16). (b) *Prdm16* expression levels were positively correlated with *Per3* expression levels in BXD strains (r=0.67, p=1.75e-11).

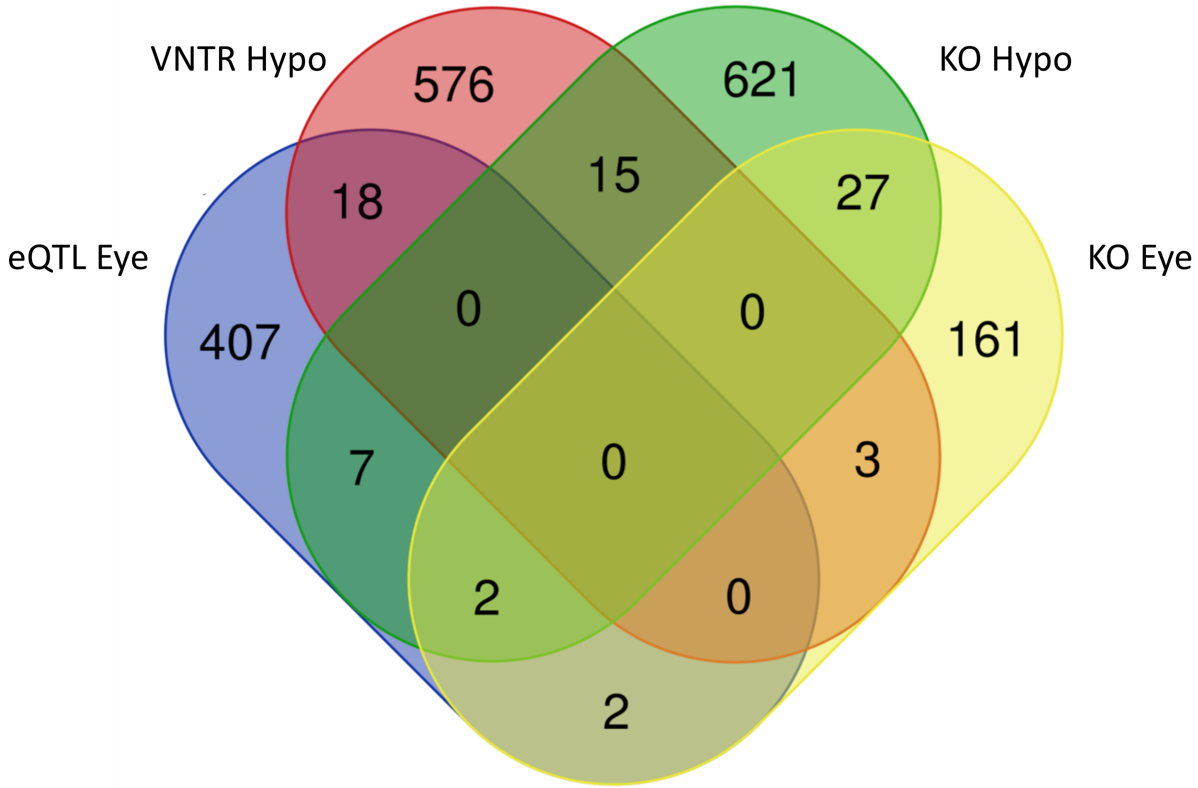

Figure S3. Venn diagram of overlap between lists of differential gene expression. 'Eye eQTL' is the list of top-500 transcripts (436 unique genes) whose expression covaries with *Per3* in the eye in BXD mice. 'VNTR45' is the list of genes differentially expressed in the hypothalamus between *Per3* 4-repeat and *Per3* 5-repeat mice (612 unique genes). 'KO Hypo' and 'KO Eye' are the lists of differentially expressed gene in the hypothalamus and eye, respectively, in the *Per3* KO mice in this study (672 and 195 unique genes, respectively).

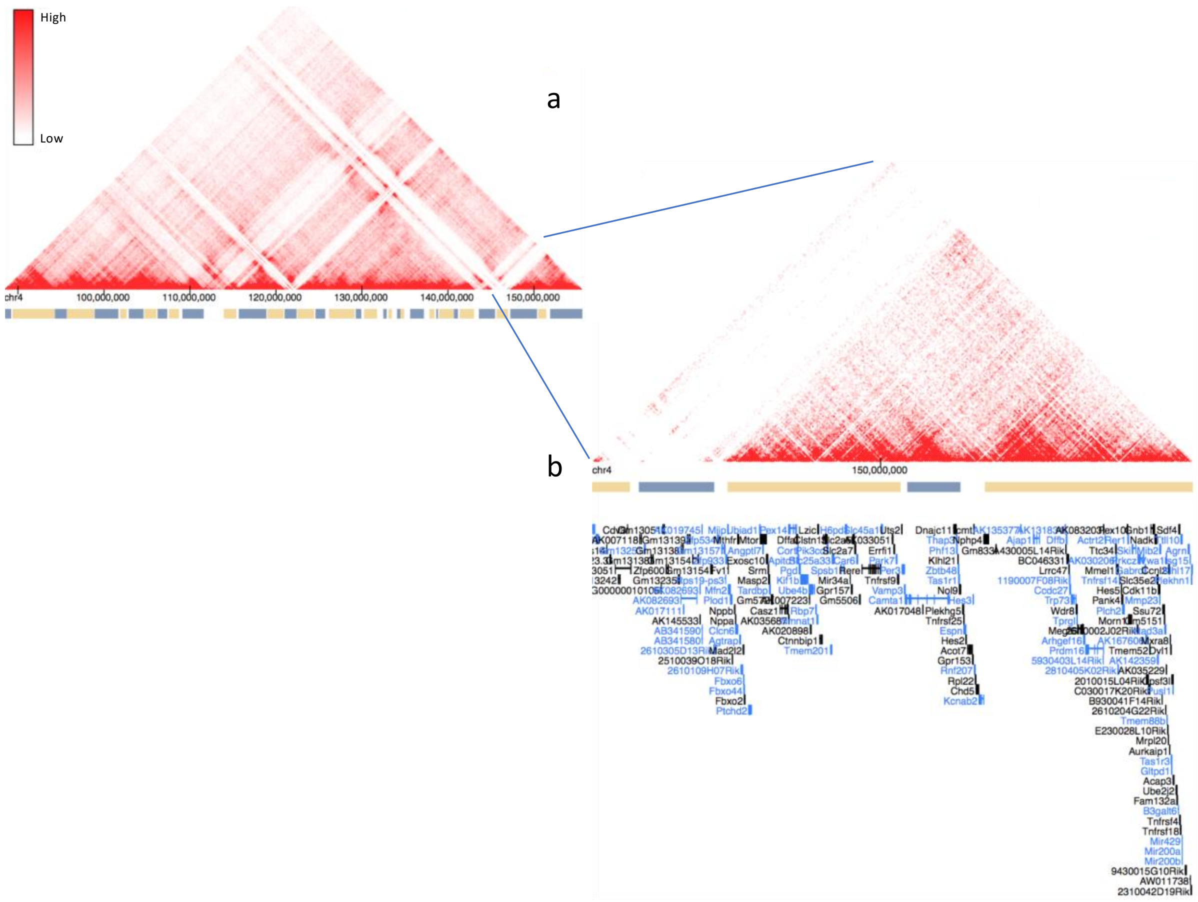

Figure S4. (a) Heatmap for Hi-C chromatin interaction scores (higher = more interaction) for chromosome 4 region 88 – 156 Mb. Predicted topologically associating domains (TADs) are indicated below in bars of alternating yellow and grey shading. (b) Hi-C chromatin interaction heatmap for the enlarged region of distal chromosome 4 at 144 – 156 Mb with three main TADs indicated below and mapped gene positions shown beneath.

Target	Forward primer	Reverse primer	Probe
Prdm16	GGAGCCATGGATCAGAAAACTC	CTTTCCGATTTCCTCTTTCATACC	TGGCCACATCCAGGCAACTGCA
Rplp0	GGGATTCGGTCTCTTCGACTAA	GCCTTTATTTCCATCTTTCTCAAATT	CCCGCCAAAGCAACCAAGTCAGC

Table S1. Primers and probes used for QPCR.

Gene list	Total overlap	Gene overlap
eQTL Eye/KO Hypo/KO Eye	2	Prdm16, Tnfrsf14
eQTL Eye/VNTR Hypo	18	Ssu72, Anks1, Mob1b, Htt, Agtrap, Mthfr, Nck1, Gpr107, Prdm2, Vps13d, Rtel1, Ppil4, Chac2, Plaa, Mrps15, Fbxo44, Cdc42, Clcn6
eQTL Eye/KO Hypo	7	Rad54l, Pon2, Phldb2, Acad11, Per3, Tbx5, Ms4a1
eQTL Eye/KO Eye	2	Ptprk, Ube2j2
VNTR Hypo/KO Hypo	15	Sep6, Akr1c12, Gm15328, E2f8, Adh1, Folr2, 4932438A13Rik, Klhl13, Foxk2, Zbtb46, Cdc6, Doc2g, Galntl6, 1500015O10Rik, Tsen2
VNTR Hypo/KO Eye	3	Plekha6, Pde4b, Pde4d
KO Hypo/KO Eye	27	Alb, Agr3, Aoc1, Sp8, Cyp2e1, S100a9, Zfp358b, 1500026H17Rik, Ltf, Kcnab2, Clstn1, Trim61, Vmn1r26, Fat3, Gm11264, Mup15, Slc2a5, Pcgf3, Mup-ps19, Gmnn, Wnk1, Gm12601, Sult1c2, Mup9, Olah, Rnf207, Reg4

Table S2. Identification of overlapping differentially expressed genes for the 'Eye eQTL' list of top-500 transcripts (436 unique genes) whose expression covaries with *Per3* in the eye in BXD mice, the 'VNTR45' list of genes differentially expressed in the hypothalamus between *Per3* 4-repeat and *Per3* 5-repeat mice (612 unique genes), and the 'KO Hypo' and 'KO Eye' lists of differentially expressed gene in the hypothalamus and eye, respectively, in the *Per3* KO mice in this study (672 and 195 unique genes, respectively).
